# Epigenetics and obesity: the devil is in the details

**DOI:** 10.1186/1741-7015-8-88

**Published:** 2010-12-21

**Authors:** Paul W Franks, Charlotte Ling

**Affiliations:** 1 Department of Clinical Sciences, Skåne University Hospital, Lund University, Malmö, Sweden; 2 Department of Nutrition, Harvard School of Public Health, Boston, MA, USA

## Abstract

Obesity is a complex disease with multiple well-defined risk factors. Nevertheless, susceptibility to obesity and its sequelae within obesogenic environments varies greatly from one person to the next, suggesting a role for gene × environment interactions in the etiology of the disorder. Epigenetic regulation of the human genome provides a putative mechanism by which specific environmental exposures convey risk for obesity and other human diseases and is one possible mechanism that underlies the gene × environment/treatment interactions observed in epidemiological studies and clinical trials. A study published in *BMC Medicine *this month by Wang *et al. *reports on an examination of DNA methylation in peripheral blood leukocytes of lean and obese adolescents, comparing methylation patterns between the two groups. The authors identified two genes that were differentially methylated, both of which have roles in immune function. Here we overview the findings from this study in the context of those emerging from other recent genetic and epigenetic studies, discuss the strengths and weaknesses of the study and speculate on the future of epigenetics in chronic disease research.

See research article: http://www.biomedcentral.com/1741-7015/8/87/abstract

## Introduction

Obesity is highly prevalent in most industrialized nations where labor-saving devices and calorically dense diets are common [1]. The impressive results from randomized clinical trials of intensive lifestyle modification on short- to medium-term weight loss [2,3] give us good reason to believe that traditional hunter-gatherer or subsistence farming lifestyles might be a panacea for the obesity epidemic. However, programs of lifestyle modification are notoriously difficult to implement and maintain on a large scale for prolonged durations, and the public health message of "eat less and exercise more" appears to have fallen on deaf ears. Thus, despite the apparently simple explanation and remedy for obesity, this knowledge is not enough. So we are saddled with a challenge, which is to unravel the mechanisms by which obesity emerges and to understand how its presence causes disease and death, with the hope that somewhere within the details hides the solution to the problem.

Although obesity is widespread, certain ethnic groups appear much more susceptible than others to the condition [4]. Striking differences in the rates of obesity are often seen between genetically distinct subpopulations, such as American Indians and people of northern European ancestry living within comparable environments, indicating that obesity may be the consequence of gene × environment interactions. More concrete evidence of such interactions has emerged from epidemiological cohort studies, most notably for the interaction between a polymorphism at the *FTO *(fat mass and obesity gene) locus and physical activity levels [5]. Nevertheless, even where rare examples of statistically reliable gene × environment interactions have emerged from epidemiological studies, causal inference is often difficult and little is revealed about the underlying mechanisms driving the interaction.

## Mechanisms of gene × environment interactions in disease

**Figure 1:**
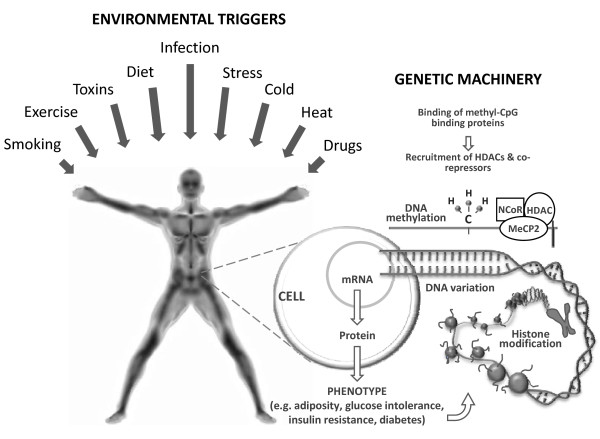
**The mechanisms that underlie observations of gene × environment interactions made in epidemiological studies (or gene × treatment interactions in clinical trials) likely involve a combination of epigenetic and transcriptional modifications**. Although environmental exposures may be the primary triggers of these perturbations, the phenotypes themselves may also feed back to trigger both epigenetic and transcriptomic events, thus modulating the expression of disease phenotypes. The figure shows a simplification of how these processes might fit together. HDAC, histone deacetyltransferase; NCor, nuclear receptor corepressor; MeCP2, methyl CpG binding protein 2.

So what mechanisms might underlie the gene × environment interactions observed in epidemiological studies or clinical trials? Clearly, the manner in which an interaction is defined and quantified will affect our ability to understand the cellular mechanisms which underlie it. However, assuming the interaction is a consequence of environmental effects (for example, smoking, physical activity, or dietary whole grains intake) on a disease trait that are dependent on the genotype of an individual, there are probably at least two closely related mechanisms that might give rise to the interaction (Figure 1).

The first involves differential transcription rates across genotypes in response to environmental stimuli. During aerobic exercise, as an example, subsets of genes, particularly those involved in oxidative energy metabolism, are upregulated [6]. If the rates of transcription or translation of these genes were to differ by genotype, one might, at a population level, observe a gene × lifestyle interaction on a clinical phenotype such as blood glucose concentrations. The second mechanism by which genes and environmental factors might interact involves epigenetic factors, such as DNA methylation and histone modifications.

The word *epigenetics *describes the phenomena of inherited changes in gene function that occur independently of changes in the nucleotide sequence [7]. Initially, it was believed that epigenetic modifications were unidirectional, but recent studies have demonstrated that the epigenome is in fact highly dynamic, changing in response to nutrient availability, physical exercise and aging, among other exposures [8-22]. While nearly all cells in the body have the same nuclear genome, different cell types have their own epigenomes, a characteristic essential for the development of cell-specific phenotypes. In differentiated mammalian cells, DNA methylation occurs primarily to cytosines in CpG-dinucleotides [23]. DNA methylation of gene promoters has also been implicated in transcriptional silencing, mainly through repressed transcription factor binding to gene promoters or by recruiting methyl-CG-binding proteins, which in turn recruit histone deacetyltransferases (HDACs) and corepressors. By virtue of the cell's capacity for histone modification, it can control its chromatin structure and either activate or suppress the transcription of its genes. Early-life nutrition represents an intriguing example of how environmentally augmented epigenetic events might affect an individual's response to metabolic load and disease susceptibility in adulthood, with numerous studies lending weight to this hypothesis [14-16,24,25]. Nevertheless, our understanding of how epigenetic events early in life influence the development of obesity and its comorbidities remains fairly rudimentary.

This month in *BMC Medicine*, Wang *et al. *[26] report a study in which they examined DNA methylation in isolated blood leukocytes and its relationship with obesity-induced immune dysfunction in seven lean and seven obese African American male adolescents. Specifically, the authors examined DNA methylation in peripheral blood leukocytes at approximately 27,000 CpG sites spread across more than 14,000 genes and compared methylation patterns between the two groups. Epigenetic studies are often constrained in their size by the high costs of the methylation chips (although this obstacle is receding as the technology becomes less expensive). This, in combination with the multitude of hypothesis tests that are performed in multiplex experiments and the corresponding procedures to correct for type 1 errors, render almost all existing epigenetic studies underpowered to detect statistically robust effects. Such is the case with the study conducted by Wang *et al.*; indeed, none of the findings from the first phase of their experiment remained statistically significant after correction for multiple testing. Despite this, the authors carried forward their most promising findings (defined as either genes yielding association *P *values ≤2 × 10^-4 ^or those with differences in DNA methylation ≥27.1%) for replication in a cohort composed of 45 obese and 46 lean individuals. In these replication analyses, two of six genes were differentially methylated in obese compared with lean individuals; the levels of DNA methylation for *UBASH3A *(ubiquitin-associated and SH3 domain-containing A) and *TRIM3 *(tripartite motif-containing 3) were higher and lower, respectively, in obese compared with lean individuals, findings that were directionally consistent with those from the first phase of the study.

Interestingly, recent genome-wide scans have implicated DNA variants proximal to *UBASH3A*, which encodes a T-cell signaling- and activation-regulating protein [27], in the development of type 1 diabetes [28]. Thus, the findings of Wang *et al.*, when placed in context with existing genetic data, suggest that *UBASH3A *may play a role in obesity-induced immune dysfunction. Intriguingly, *TRIM3*, which belongs to the superfamily of TRIM proteins, is also involved in immune response [29], which may explain why differential methylation patterns of these genes were visible in blood leukocytes.

The study by Wang *et al. *is small and probably underpowered to detect all but the largest differences in DNA methylation. It may be, therefore, that with a larger sample size, statistically significant differences in DNA methylation patterns for many other genes that are smaller in magnitude than for *UBASH3A *and *TRIM1 *would be observable. The detection of other CpG sites and genes might also be facilitated by the application of methods that afford greater genomic coverage, such as deep sequencing combined with either bisulfite-treated DNA or immunoprecipitation of methylated DNA.

While peripheral blood leukocytes are attractive for epigenetic studies, not least because they can be easily obtained, there are many other cell types and tissues involved in the pathogenesis of obesity and its comorbidities, such as adipose tissue, skeletal muscle and hypothalamus, the examination of which could be well worthwhile in the context of epigenetics. Along these lines, Bouchard *et al. *[17] recently provided some of the first evidence that DNA methylation in adipose tissue differs in people who respond well and those who respond poorly to caloric restriction for weight loss. Elsewhere, a high-fat diet lasting 5 days was shown to affect DNA methylation of a major transcriptional coactivator (peroxisome proliferator-activated receptor-γ coactivator 1-α) of genes involved in oxidative energy metabolism [13]. Furthermore, in studies examining early life overnutrition and obesity, DNA methylation of the hyperthalamically expressed pro-opiomelanocortin promoter, which plays an important role in hunger and satiation [30], was observed. While these studies imply that epigenetic modifications cause obesity, metabolic disease and its complications, it is virtually impossible to establish whether changes in methylation precede the development of obesity or *vice versa*. Indeed, these relationships may not be causal at all, but consequences of confounding by factors correlated with obesity and DNA methylation, such as physical inactivity, nutrition or smoking.

Elucidating causal relationships between DNA methylation and obesity will be necessary if observations of the nature described by Wang *et al. *are to be of clinical value. Inevitably, this probably means that randomized, controlled trials of weight loss or weight gain interventions, where DNA methylation patterns are assessed before and after the intervention and subsequently compared between the treatment and control arms of the trial, are required. It will also be necessary to examine whether changes in DNA methylation correspond with changes in gene transcription and/or translation, as well as more distal, clinically relevant phenotypes. Assessing these relationships in diverse tissues and cell types and in different demographic groups and environmental contexts will further advance our understanding of how epigenetic events affect an individual's predisposition to obesity, or how obesity impacts the epigenome. Whether changes in DNA methylation are associated with obesity-induced histone modifications of the same genes (that is, whether DNA methylation coincides with the presence of closed histone marks) represents another interesting but as yet unanswered question.

## Concluding remarks

In summary, Wang *et al.*'s study provides tentative evidence that DNA methylation at two loci, *UBASH3A *and *TRIM3*, may be implicated in the pathogenesis of obesity. Replication of these findings in independent settings will be necessary to ensure that these findings are true positives, and to fairly conclude that the relationships are causal will require appropriately designed experimental studies. Because of these and other hurdles facing the field of epigenetics, identifying a meaningful clinical application for epigenetics in the prevention or treatment of obesity is likely to remain more vision than reality for some time to come.

## Competing interests

The authors declare that they have no competing interests.

## Authors' contributions

PWF wrote the first draft of the article. Both authors contributed to the development of the draft.

## Pre-publication history

The pre-publication history for this paper can be accessed here:

http://www.biomedcentral.com/1741-7015/8/88/prepub

## References

[B1] World Health Organization Global Health Risks: Mortality and Burden of Disease Attributable to Selected Major Risk Factors 2009 Geneva, Switzerland: World Health Organization

[B2] Knowler WC Barrett-Connor E Fowler SE Hamman RF Lachin JM Walker EA Nathan DM Reduction in the incidence of type 2 diabetes with lifestyle intervention or metformin N Engl J Med 2002 346 393 403 10.1056/NEJMoa012512 11832527 PMC1370926

[B3] Tuomilehto J Lindstrom J Eriksson JG Valle TT Hamalainen H Ilanne-Parikka P Keinanen-Kiukaanniemi S Laakso M Louheranta A Rastas M Salminen V Aunola S Cepaitis Z Moltchanov V Hakumaki M Mannelin M Martikkala V Sundvall J Uusitupa M Finnish Diabetes Prevention Study Group Prevention of type 2 diabetes mellitus by changes in lifestyle among subjects with impaired glucose tolerance N Engl J Med 2001 344 1343 1350 10.1056/NEJM200105033441801 11333990

[B4] Ogden CL Carroll MD Curtin LR McDowell MA Tabak CJ Flegal KM Prevalence of overweight and obesity in the United States, 1999-2004 JAMA 2006 295 1549 1555 10.1001/jama.295.13.1549 16595758

[B5] Andreasen CH Stender-Petersen KL Mogensen MS Torekov SS Wegner L Andersen G Nielsen AL Albrechtsen A Borch-Johnsen K Rasmussen SS Clausen JO Sandbaek A Lauritzen T Hansen L Jorgensen T Pedersen O Hansen T Low physical activity accentuates the effect of the FTO rs9939609 polymorphism on body fat accumulation Diabetes 2008 57 95 101 10.2337/db07-0910 17942823

[B6] Franks PW Loos RJ PGC-1alpha gene and physical activity in type 2 diabetes mellitus Exerc Sport Sci Rev 2006 34 171 175 10.1249/01.jes.0000240021.92254.23 17031255

[B7] Bird A Perceptions of epigenetics Nature 2007 447 396 398 10.1038/nature05913 17522671

[B8] McGee SL Hargreaves M Exercise and myocyte enhancer factor 2 regulation in human skeletal muscle Diabetes 2004 53 1208 1214 10.2337/diabetes.53.5.1208 15111488

[B9] McGee SL Hargreaves M Exercise and skeletal muscle glucose transporter 4 expression: molecular mechanisms Clin Exp Pharmacol Physiol 2006 33 395 399 10.1111/j.1440-1681.2006.04362.x 16620308

[B10] McGee SL Howlett KF Starkie RL Cameron-Smith D Kemp BE Hargreaves M Exercise increases nuclear AMPK α_2 _in human skeletal muscle Diabetes 2003 52 926 928 10.2337/diabetes.52.4.926 12663462

[B11] McGee SL Sparling D Olson AL Hargreaves M Exercise increases MEF2- and GEF DNA-binding activity in human skeletal muscle FASEB J 2006 20 348 349 16368714 10.1096/fj.05-4671fje

[B12] McGee SL van Denderen BJ Howlett KF Mollica J Schertzer JD Kemp BE Hargreaves M AMP-activated protein kinase regulates GLUT4 transcription by phosphorylating histone deacetylase 5 Diabetes 2008 57 860 867 10.2337/db07-0843 18184930

[B13] Brøns C Jacobsen S Nilsson E Rönn T Jensen CB Storgaard H Poulsen P Groop L Ling C Astrup A Vaag A Deoxyribonucleic acid methylation and gene expression of PPARGC1A in human muscle is influenced by high-fat overfeeding in a birth-weight-dependent manner J Clin Endocrinol Metab 2010 95 3048 3056 20410232 10.1210/jc.2009-2413

[B14] Lillycrop KA Phillips ES Jackson AA Hanson MA Burdge GC Dietary protein restriction of pregnant rats induces and folic acid supplementation prevents epigenetic modification of hepatic gene expression in the offspring J Nutr 2005 135 1382 1386 15930441 10.1093/jn/135.6.1382

[B15] Lillycrop KA Phillips ES Torrens C Hanson MA Jackson AA Burdge GC Feeding pregnant rats a protein-restricted diet persistently alters the methylation of specific cytosines in the hepatic PPAR alpha promoter of the offspring Br J Nutr 2008 100 278 282 10.1017/S0007114507894438 18186951 PMC2564112

[B16] Lillycrop KA Slater-Jefferies JL Hanson MA Godfrey KM Jackson AA Burdge GC Induction of altered epigenetic regulation of the hepatic glucocorticoid receptor in the offspring of rats fed a protein-restricted diet during pregnancy suggests that reduced DNA methyltransferase-1 expression is involved in impaired DNA methylation and changes in histone modifications Br J Nutr 2007 97 1064 1073 10.1017/S000711450769196X 17433129 PMC2211425

[B17] Bouchard L Rabasa-Lhoret R Faraj M Lavoie ME Mill J Pérusse L Vohl MC Differential epigenomic and transcriptomic responses in subcutaneous adipose tissue between low and high responders to caloric restriction Am J Clin Nutr 2010 91 309 320 10.3945/ajcn.2009.28085 19939982

[B18] Fraga MF Ballestar E Paz MF Ropero S Setien F Ballestar ML Heine-Suner D Cigudosa JC Urioste M Benitez J Boix-Chornet M Sanchez-Aguilera A Ling C Carlsson E Poulsen P Vaag A Stephan Z Spector TD Wu YZ Plass C Esteller M Epigenetic differences arise during the lifetime of monozygotic twins Proc Natl Acad Sci USA 2005 102 10604 10609 10.1073/pnas.0500398102 16009939 PMC1174919

[B19] Ling C Groop L Epigenetics: a molecular link between environmental factors and type 2 diabetes Diabetes 2009 58 2718 2725 10.2337/db09-1003 19940235 PMC2780862

[B20] Ling C Del Guerra S Lupi R Rönn T Granhall C Luthman H Masiello P Marchetti P Groop L Del Prato S Epigenetic regulation of PPARGC1A in human type 2 diabetic islets and effect on insulin secretion Diabetologia 2008 51 4 615 22 10.1007/s00125-007-0916-5 18270681 PMC2270364

[B21] Ronn T Poulsen P Hansson O Holmkvist J Almgren P Nilsson P Tuomi T Isomaa B Groop L Vaag A Ling C Age influences DNA methylation and gene expression of COX7A1 in human skeletal muscle Diabetologia 2008 51 1159 1168 10.1007/s00125-008-1018-8 18488190

[B22] Ling C Poulsen P Simonsson S Ronn T Holmkvist J Almgren P Hagert P Nilsson E Mabey AG Nilsson P Vaag A Groop L Genetic and epigenetic factors are associated with expression of respiratory chain component NDUFB6 in human skeletal muscle J Clin Invest 2007 117 3427 3435 10.1172/JCI30938 17948130 PMC2030455

[B23] Lister R Pelizzola M Dowen RH Hawkins RD Hon G Tonti-Filippini J Nery JR Lee L Ye Z Ngo QM Edsall L Antosiewicz-Bourget J Stewart R Ruotti V Millar AH Thomson JA Ren B Ecker JR Human DNA methylomes at base resolution show widespread epigenomic differences Nature 2009 462 315 322 10.1038/nature08514 19829295 PMC2857523

[B24] Tobi EW Lumey LH Talens RP Kremer D Putter H Stein AD Slagboom PE Heijmans BT DNA Methylation differences after exposure to prenatal famine are common and timing- and sex-specific Hum Mol Genet 2009 18 4046 4053 10.1093/hmg/ddp353 19656776 PMC2758137

[B25] Heijmans BT Tobi EW Stein AD Putter H Blauw GJ Susser ES Slagboom PE Lumey LH Persistent epigenetic differences associated with prenatal exposure to famine in humans Proc Natl Acad Sci USA 2008 105 17046 17049 10.1073/pnas.0806560105 18955703 PMC2579375

[B26] Wang X Zhu H Sneider H Su S Munn D Harshfiels G Maria BL Dong Y Treiber F Gutin B Shi H Obesity related methylation changes in DNA of peripheral blood leukocytes BMC Med 2010 8 87 10.1186/1741-7015-8-14 21176133 PMC3016263

[B27] Carpino N Turner S Mekala D Takahashi Y Zang H Geiger TL Doherty P Ihle JN Regulation of ZAP-70 activation and TCR signaling by two related proteins, Sts-1 and Sts-2 Immunity 2004 20 37 46 10.1016/S1074-7613(03)00351-0 14738763

[B28] Barrett JC Clayton DG Concannon P Akolkar B Cooper JD Erlich HA Julier C Morahan G Nerup J Nierras C Plagnol V Pociot F Schuilenburg H Smyth DJ Stevens H Todd JA Walker NM Rich SS Genome-wide association study and meta-analysis find that over 40 loci affect risk of type 1 diabetes Nat Genet 2009 41 703 707 10.1038/ng.381 19430480 PMC2889014

[B29] Ozato K Shin DM Chang TH Morse HC TRIM family proteins and their emerging roles in innate immunity Nat Rev Immunol 2008 8 849 860 10.1038/nri2413 18836477 PMC3433745

[B30] Plagemann A Harder T Brunn M Harder A Roepke K Wittrock-Staar M Ziska T Schellong K Rodekamp E Melchior K Dudenhausen JW Hypothalamic proopiomelanocortin promoter methylation becomes altered by early overfeeding: an epigenetic model of obesity and the metabolic syndrome J Physiol 2009 587 4963 4976 10.1113/jphysiol.2009.176156 19723777 PMC2770159

